# Social Support and Functional Dentition Among English Older Adults

**DOI:** 10.3390/dj13120554

**Published:** 2025-11-24

**Authors:** Fatimah Alobaidi, Wael Sabbah

**Affiliations:** Faculty of Dentistry, Oral & Craniofacial Sciences, 2nd Floor Dental Institute Extension, King’s College London, Denmark Hill, London SE5 9RS, UK; wael.sabbah@kcl.ac.uk

**Keywords:** adults, aged, dentition, social support

## Abstract

**Objectives**: The aim of this study was to examine the association between social support and functional dentition among older adults in England. **Methods**: A cross-sectional analysis was conducted using data from Wave 7 (2014/15) of the English Longitudinal Study of Ageing (ELSA). Functional dentition, defined as having 20 or more natural teeth was the outcome. Positive and negative social support scores were included as exposures. Smoking and socioeconomic factors were included as covariates. Poisson regression models with robust variance estimators were used to estimate prevalence ratios (PRs) for functional dentition, adjusting sequentially for demographic, socioeconomic, and behavioural factors. **Results**: A total of 6457 participants were included. Most participants in the sample were female, white, had less than O-Level education, were in higher wealth groups, had never smoked, and reported having functional dentition. The majority reported higher levels of positive social support and lower levels of negative social support. In the fully adjusted Poisson model, each one-point increase in positive social support score was associated with a 1% higher prevalence of functional dentition (PR = 1.01, 95% CI: 1.01–1.01), while negative social support was not significantly associated with functional dentition after adjustment. **Conclusions**: Positive social support was significantly associated with maintaining functional dentition among older English adults, independent of socioeconomic and behavioural factors. However, as this study was cross-sectional and based on self-reported data, causal relationships cannot be inferred. Interventions in community and healthcare settings that encourage social engagement, reduce isolation, and integrate oral health promotion within wider social care strategies may contribute to preserving functional dentition and reducing oral health inequalities. Future research should explore how these relationships evolve across the life course and interact with behavioural and socioeconomic determinants of health.

## 1. Introduction

As the population ages, social inequalities in oral health persist among older adults. Lower social circumstances are more common in this population, placing them at higher risk for both general and oral health problems [1]. Social support can be defined as the emotional and physical assistance provided by social connections and ties [2,3]. This support may be positive, such as encouragement and self-appraisal, or negative, such as excessive criticism [4]. The nature of this support can influence oral health outcomes through shaping social norms, facilitating dissemination of health-related knowledge, and enhancing resilience to stress [5]. For instance, negative social connections such as isolation or weak relationships are linked to the absence of functional dentition, reflected in greater tooth loss or impaired oral function [6,7]. Functional dentition, defined as having at least 20 natural teeth [8], and is particularly important because its absence can reduce quality of life by compromising masticatory function, nutrient intake, and social interaction [9,10].

Previous studies examining the impact of social support on tooth loss have reported that lower levels of support are associated with increased tooth loss [11,12]. One study investigated the relationship between social support and multiple oral health conditions in an Australian population and found that lower social support was linked to poor outcomes, including having fewer than 20 teeth [13]. However, this study focused on only positive social support such as emotional encouragement and practical assistance, which can promote healthier behaviours and better oral hygiene practices. In contrast, negative social support, which includes criticism, conflict, or excessive demands within social relationships, may increase stress and discourage health-promoting behaviours [5]. Another study explored the association between social support and edentulism in an English population and reported that low levels of social support were a significant predictor of edentulism [14], but it did not differentiate between these opposing dimensions. Distinguishing between positive and negative social support is therefore important for understanding their distinct pathways and potential impacts on oral health outcomes.

These findings highlight the important role of social support in oral health outcomes. Although several studies have examined this relationship, to the best of our knowledge, no study has specifically explored the association between positive and negative social support and functional dentition among English older adults. Moreover, socioeconomic factors such as education and wealth, as well as behaviours such as smoking, may influence this association, either strengthening or weakening the impact of social support. Therefore, the aim of this study was to investigate the independent associations of positive and negative social support with functional dentition, adjusting for socioeconomic and behavioural covariates among English older adults.

## 2. Materials and Methods

### 2.1. Study Design and Participants

This study is a cross-sectional study based on a secondary data analysis of Wave 7 (2014/15) from the English Longitudinal Study of Ageing ELSA [15]. This comprehensive dataset collects multiple information on participants aged 50 and older. The initial ELSA cohort was derived from the Health Survey for England (HSE), conducted in 1998, 1999, and 2001. ELSA survey employed a multi-stage stratified sampling design, dividing the population into strata and selecting participants at random using computer-generated methods to ensure representativeness. As the ELSA data are publicly available, no additional ethical approval was required for the present analysis. For reference, the original ELSA study received ethical approval from the Multicentre Research and Ethics Committee (MREC) under the code MREC/01/2/91. The first Wave received approval on 7 February 2002, while Wave 7 (the wave used in the study) received approval on 28 November 2013.Data regarding social, physical, and mental health were collected using Computer-Assisted Personal Interviews (CAPI) followed by Self-Completion Questionnaires (SCQ). Oral health information was gathered in three Waves starting from Wave 3 (2006/07), Wave 5 (2010/11), and Wave 7 (2014/15). Wave 7 was selected because it was the only wave that collected information on functional dentition, with 9666 participants completing the interviews in person.

### 2.2. Variables

The outcome was functional dentition, defined according to the World Health Organization (WHO) criteria “having at least twenty natural teeth that are both functionally effective and aesthetically acceptable throughout a person’s life, without the need for prosthetic replacement.” [8]. Functional dentition was assessed during CAPI where participants were asked “How many natural teeth have you got?” with response options: “none at all, between 1 and 9 natural teeth, between 10 and 19 natural teeth, 20 or more natural teeth”. The variable was dichotomised as fewer than 20 teeth (No) vs. 20 teeth or more (Yes).

The main exposure was social support, collected during SCQ. Participants were asked about their relationship with their spouse, children, family, and friends. Positive social support was assessed with questions on “*How much does [the person] really understand the way you feel about things?”, “How much can you rely on [them] if you have a serious problem?”,* and *“How much can you open up to [them] if you need to talk about your worries?”.* Responses were measured on a 4-item scale (from 0–3) and summed to create a total score range from 0 to 36. Negative social support was assessed with questions *“How much does [the person] criticise you?”, “How much do they let you down when you are counting on them?”,* and *“How much do they get on your nerves?”.* Responses were scored in the same way (from 0–3) and summed into a score ranging from 0 to 28. The creation of these composite scores followed the same approach used in previous studies employing ELSA data and similar social support measures, ensuring comparability and methodological consistency [16]. These scales have previously shown acceptable internal consistency in ELSA data (Cronbach’s α = 0.81 for positive support and α = 0.79 for negative support in one subsample) [17]. Although full factor—analytic loadings have not been published in ELSA documentation, the item structure has been used consistently in previous studies [16].

Multiple covariates were also included. Smoking status was assessed using two self-reported questions: “Do you smoke cigarettes at all nowadays?” and “Have you ever smoked?” with yes/no response options. Based on participants’ responses, smoking was categorised into three groups: never smokers (those who reported never having smoked), former smokers (those who had smoked in the past but no longer did), and current smokers (those who reported currently smoking). Education was used as collected in the survey (less than O-levels, O-levels, higher education or A levels). O-Levels are comparable to high school diplomas, whereas A-Levels correspond to advanced high school diplomas or pre-university qualifications [18]. Sex was recorded as (male or female). Age was used as a continuous variable. Ethnicity was available in the dataset as white vs. non-white and was used as such. Wealth quantiles were created in the survey based on quintiles of total non-pension wealth, ranging from 1 (poorest) to 5 (wealthiest). Total wealth was calculated by summing the value of housing, financial assets, and physical possessions owned by the household, while excluding debts [19].

### 2.3. Statistical Analysis

Poisson regression models with robust variance estimators were used to investigate the associations between social support and functional dentition, providing prevalence ratios (PRs) and 95% confidence intervals (CIs) as interpretable measures of relative prevalence [20]. Three models were constructed, crude model (unadjusted), partially adjusted, and fully adjusted model. The partially adjusted model included demographic factors (age, sex and ethnicity), and socioeconomic factors (education and wealth). The fully adjusted model further adjusted for smoking to see how smoking influences the relationship between social support and functional dentition. Multicollinearity among predictors was evaluated using uncentered variance inflation factors (VIFs) derived from a linear regression on the unweighted sample, as VIFs cannot be directly calculated for survey-weighted Poisson regression. The overall fit and robustness of the Poisson models were assessed using Wald F tests. As a sensitivity analysis, logistic regression models were estimated using the same variable specifications to assess the consistency of findings. A further sensitivity analysis excluded edentulous participants to verify that the observed associations were not driven by those without natural teeth.

All analysis was conducted in STATA software version 19. Stata survey commands were applied in all analyses, incorporating cross-sectional sample weights to account for the survey design. A complete-case analysis approach was used, whereby participants with missing data on any study variable were excluded from the analysis.

## 3. Results

After excluding 3209 participants due to missing data, 6457 participants were included in the analysis (Supplementary Figure S1). Table 1 displays the characteristics of the included and excluded samples. Functional dentition was slightly more common among the excluded sample (70.9%) than the included sample (66.7%). The included sample had a mean age of 66 years, slightly higher than the excluded sample (63 years). Most participants in both samples were White and female. Positive and negative social support scores were similar across groups. Compared with excluded participants, those included in the analysis had higher educational attainment and wealth, indicating more favourable sociodemographic characteristics.

Table 2 displays participant characteristics by functional dentition status. Participants with functional dentition were younger (mean age 63 years) than those without functional dentition (mean age 72 years). Both groups were predominantly White and had similar gender distributions, with no substantial differences between males and females. Higher education and wealth were associated with functional dentition, with 80.5% of participants with higher education and 82.3% of participants in the highest wealth quintile having functional dentition. Positive social support was slightly higher among participants with functional dentition, whereas negative social support was slightly lower. Smoking status showed clear differences: current smokers were more common among participants without functional dentition (50.1%) compared with those with functional dentition (49.9%), and former smokers were more prevalent in the functional dentition group. These comparisons indicate differences in characteristics between the two groups but do not imply prediction or causation.

Table 3 presents the associations from the Poisson regression models estimating prevalence ratios (PRs) for having functional dentition. The overall fit of the Poisson regression models was assessed using Wald F tests. Both the partially adjusted and fully adjusted models demonstrated statistically significant overall fit (partially adjusted model: F(11, 8112) = 122.54, *p* < 0.001; fully adjusted model: F(13, 8110) = 109.62, *p* < 0.001), indicating that the included predictors collectively explained a significant portion of variation in functional dentition.

In the crude (unadjusted) model, being non-white, younger, having higher educational attainment, and greater wealth were all significantly associated with a higher prevalence of functional dentition. Positive social support was positively associated with functional dentition (PR = 1.01, 95% CI: 1.01–1.02), and negative social support showed a positive association (PR = 1.01, 95% CI: 1.01–1.01). Compared with never smokers, former smokers had a higher prevalence (PR = 1.14, 95% CI: 1.02–1.27), while current smokers had a substantially lower prevalence of functional dentition (PR = 0.73, 95% CI: 0.67–0.79).

In the partially adjusted model, which controlled for demographic, socioeconomic, and social variables, education, wealth, age, and positive social support remained significant predictors. The magnitude of associations was somewhat attenuated compared to the crude model. Positive social support remained positively associated (PR = 1.01, 95% CI: 1.01–1.01), while negative social support became non-significant.

In the fully adjusted model, which further included smoking, most associations persisted. Higher education and wealth remained strongly associated with greater prevalence of functional dentition, while age continued to show an inverse association (PR = 0.97, 95% CI: 0.96–0.97). Positive social support retained a significant positive association (PR = 1.01, 95% CI: 1.01–1.01), while negative social support remained non-significant. Regarding smoking, current smokers had a markedly lower prevalence of functional dentition (PR = 0.74, 95% CI: 0.69–0.81), while former smokers did not differ significantly from never smokers.

Multicollinearity among predictors was evaluated using uncentered variance inflation factors (VIFs) from a linear regression on the unweighted sample, including the same variables as the main model (Supplementary Table S1). Linear regression was used rather than Poisson regression because VIF calculations are not directly supported after survey-weighted models. VIFs ranged from 1.16 (smoking) to 16.28 (age), with a mean of 7.13. Most variables, including negative social support, education, and smoking, exhibited low collinearity (VIF < 5). Higher VIFs were observed for age, sex, wealth, and positive social support, indicating some correlation among these predictors; however, these levels were not considered sufficient to compromise the interpretation of the model coefficients.

To assess the robustness of these findings, a sensitivity analysis was conducted using survey-weighted logistic regression models (Supplementary Table S2). The direction and significance of associations were consistent with the main Poisson regression results, although effect sizes were larger, reflecting that odds ratios tend to overestimate associations when outcomes are common.

A further sensitivity analysis excluding edentulous participants showed consistent findings with the main model (Supplementary Table S3). Higher education, greater wealth, younger age, and higher positive social support were associated with a higher prevalence of functional dentition, while current smoking remained strongly inversely associated. The exclusion of edentulous participants did not materially change the direction or significance of associations, supporting the robustness of the main results.

## 4. Discussion

This study explored the association between social support and functional dentition. The findings revealed that individuals with higher positive social support (such as understanding and reliability) were more likely to have functional dentition, while negative social support was not significantly associated with functional dentition after adjusting for socioeconomic and behavioural factors. Adding a health behaviour that is linked to oral health such as smoking slightly attenuated the associations, but the effects remained statistically significant. Sensitivity analyses, including logistic regression and the exclusion of edentulous participants, confirmed the robustness of the findings. Although the effect sizes for positive social support were modest per one-point increase, it remains meaningful from a public health perspective. Given that the social support scales cover a wide range, cumulative differences between individuals with consistently high versus low support could translate into substantial variations in oral health outcomes at the population level. Thus, even small improvements in social connectedness may contribute to the maintenance of functional dentition when reinforced through social and behavioural interventions.

One previous study has confirmed these results. One study among Australian adults revealed that individuals with lower social support had poor oral health including having less than 21 teeth [13]. However, the association with functional dentition was weak. One possible explanation is that this study focused on the presence of positive social support, while in reality, the balance of both positive and negative relationship dynamics probably plays a stronger role. Several studies indicated that both the positive and negative aspects of social relationship dynamics, in interaction with socioeconomic conditions, are likely to have a stronger role in maintaining teeth than either dimension alone [6,7,14,21].

Other studies have explored the effect of social support on tooth loss and revealed similar findings. A recent systematic review and meta-analysis examined the relationship between social relationships and tooth loss in older adults [7]. The pooled findings suggested that weaker social relationships were associated with a modestly higher likelihood of having fewer teeth or being edentulous. The consistent direction and magnitude of associations across the main Poisson and sensitivity analyses in current study further reinforce this relationship, suggesting that the link between social connectedness and oral health is robust across model specifications and analytic assumptions. This growing body of evidence supports the current study’s findings, underscoring the consistent association between social connectedness and oral health outcomes across different populations and settings. The review also highlighted the possibility of reverse causation, as losing teeth might reduce social interaction, not just the other way around [7].

In the current analysis, negative social support was associated with poorer oral health in the crude model but became non-significant after accounting for socioeconomic and behavioural factors. This pattern likely reflects confounding or suppression effects. In the unadjusted model, individuals with lower socioeconomic status may experience both higher negative interactions and poorer oral health. Once wealth, education, and smoking were controlled for, the independent association between negative support and tooth loss diminished. This suggests that socioeconomic position may confound or suppress the true relationship between negative social support and oral health. Without considering socioeconomic status and other confounders, both positive and negative support appeared to be associated with a protective effect on dentition; however, once these conditions were taken into account, only positive social support remained a significant predictor. Previous evidence has demonstrated that positive and negative interactions can have different and independent associations with health outcomes [22,23]. Previous studies have similarly confirmed that socioeconomic factors play a central role in shaping the relationship between social support and tooth loss, with adjustment for education, income, and wealth consistently attenuating or modifying the observed associations [7,13,14]. It has also been suggested that although social support is an important determinant of oral health, it should be considered within a broader multifactorial framework that incorporates behavioural and socioeconomic influences [7].

Smoking is an important confounder in the relationship between social support and functional dentition. On one hand, smoking is a well-established risk factor for tooth loss through its impact on periodontal disease and oral tissue healing [24,25,26]. On the other hand, social support may influence smoking behaviours, with higher levels of support associated with lower likelihood of smoking initiation and greater success in cessation [27], while weaker or negative social ties may reinforce smoking as a coping mechanism [28]. Thus, part of the association between social support and tooth retention may be explained by differences in smoking behaviours across social groups. In the current analysis, smoking was significantly associated with functional dentition but did not affect the association between social support and functional dentition. Current smokers had a markedly lower prevalence of functional dentition, while former smokers did not differ significantly from never smokers after adjustment, suggesting that those who stopped smoking long ago resemble never smokers after accounting for confounders [29].

Socioeconomic factors are strongly and consistently linked to the maintenance of functional dentition. Similar to the study findings, individuals with higher education, income, and wealth are more likely to retain a greater number of natural teeth into older age, while those from disadvantaged backgrounds face substantially higher risks of severe tooth loss and edentulism [30,31,32]. Moreover, socioeconomic inequalities determine the affordability and availability of restorative and prosthetic treatments, meaning that disadvantaged individuals not only lose more teeth but are also less able to replace them [33]. Functional dentition, therefore, can be seen as a marker of social and economic inequality, capturing disparities in access to care and health-promoting resources [34].

Even though this study did not examine the potential bidirectional nature of the association, it is important to acknowledge that the relationship between oral health and social support may not be entirely unidirectional. Poor oral health could also influence levels of social support. Individuals experiencing tooth loss or reduced oral function may face difficulties in eating, speaking, or smiling confidently, which can lead to embarrassment, lower self-esteem, and social withdrawal [35,36]. These challenges may, in turn, reduce opportunities for positive social interactions and increase the likelihood of experiencing negative or strained relationships [37,38]. Previous research has shown that poor oral health is linked to lower levels of social participation, social isolation, and diminished perceived support among older adults [39,40]. Thus, individuals with compromised oral health might not only receive less emotional or practical support but may also perceive their existing relationships more negatively. Recognising this possibility of reverse causality underscores the need for longitudinal studies to clarify the direction of the relationship and to assess whether interventions that improve oral health could also enhance social connectedness and wellbeing.

This study has some limitations that should be acknowledged. First, although the data were drawn from a longitudinal survey, information on the number of teeth was only available in Wave 7. As a result, the analysis relied on cross-sectional data, which limits the ability to infer causality or establish temporality. Second, the study relied on self-reported measures, which are subject to potential biases, including recall and social desirability bias. However, self-reported data are widely used in epidemiological research as they are cost-effective, practical, and generally well accepted. Third, although multicollinearity was checked and found to be within acceptable limits, residual confounding cannot be ruled out, as some relevant factors such as diet, comorbidities, or dental-care access were not included in the analysis. Fourth, the study could not investigate differences across specific ethnic groups, as detailed ethnicity information was withheld from the dataset to protect participant confidentiality. Finally, potential correlations between variables such as education and wealth, or between positive and negative social support, may still have influenced the observed associations.

However, the current study is the first to explore positive and negative social support in relation to functional dentition among older English population, highlighting its contribution to the evidence base. Additionally, the study used a comprehensive database which included validated instruments and applied sampling weights, reducing the chances of systematic errors (selection bias) and enhancing the generalisability of the findings.

The findings of this study highlight the importance of strengthening social support networks to promote better oral health outcomes in later life. Public health initiatives that encourage social participation, reduce isolation, and foster positive relationships may contribute to the maintenance of functional dentition among older adults. For instance, community-based programmes that promote intergenerational interaction, peer-support networks, and social clubs for older adults could help enhance social engagement while indirectly supporting oral health behaviours such as regular dental attendance and good oral hygiene practices. To operationalize these initiatives within dental or primary care workflows, practitioners could incorporate brief social connectedness assessments during routine health or dental visits to identify older adults at risk of social isolation. Patients identified as socially isolated could be referred to local community programs or peer-support groups, integrated into existing social care services. Dental practices could also collaborate with community centres to offer combined oral health education and social activities, ensuring that social and oral health support is delivered in a coordinated and feasible manner. Additionally, training primary care and dental staff to recognize the links between social support and oral health could facilitate targeted interventions for at-risk populations. Policies that integrate oral health promotion within broader social care strategies may further reduce inequalities by targeting individuals with weaker social ties. Integrating oral health promotion within broader social care and ageing strategies, particularly those aimed at strengthening social support, could help address inequalities and promote healthy ageing. Future research should further explore how different dimensions of social support, both positive and negative, influence oral health trajectories, while also considering the interaction with socioeconomic and behavioural factors across the life course.

## 5. Conclusions

Positive social support was independently associated with functional dentition among older English adults. Smoking and socioeconomic factors also influenced the likelihood of maintaining functional dentition. Promoting initiatives that foster positive social connections may support oral health and contribute to healthy ageing.

## Figures and Tables

**Table 1:** Characteristics of the included and excluded sample of ELSA wave 7 (2014/15).

	Included Sample Wave 7 (2014/15) (n = 6457)			Excluded Sample Wave 7 (2014/15) (n = 3209)		
Variables	^a^ n	%, Mean	95% CI	%, Mean	95% CI	^b^ *p*-Value
Functional dentition						
No	2361	33.25%	(31.99, 34.54)	29.22%	(29.04, 32.67)	
Yes	4222	66.75%	(65.47, 68.01)	70.88%	(65.33, 71.67)	0.002
Age	6583	66.13	(65.81, 66.35)	63.24	(63.17, 64.86)	<0.001
Gender						
Male	2906	47.04%	(45.61, 48.48)	49.48%	(49.5, 49.5)	
Female	3677	52.96%	(51.52, 54.39)	50.52%	(48.82, 55.04)	0.071
Ethnicity						
White	6405	95.77%	(94.92, 96.48)	87.85%	(86.09, 90.79)	
Non-white	178	4.23%	(3.52, 5.08)	12.25%	(9.21, 13.91)	<0.001
Education						
Less than o-level	2563	40.89%	(39.5, 42.29)	53.00%	(50.10, 56.47)	
O-level	1905	30.18%	(28.85, 31.55)	25.25%	(21.76, 27.36)	
Higher than A lever	2115	28.93%	(27.66, 30.23)	22.85%	(19.75, 24.98)	<0.001
Wealth						
Lowest	980	17.86%	(16.74, 19.04)	29.44%	(26.48, 32.58)	
Second lowest	1176	20.38%	(19.18, 21.63)	18.45%	(17.29, 22.43)	
Middle	1386	20.08%	(18.99, 21.21)	19.77%	(17.44, 22.34)	
Second highest	1494	20.66%	(19.56, 21.79)	16.91%	(13.94, 19.01)	
Highest	1548	21.03%	(19.93, 22.517)	15.48%	(12.60, 17.16)	<0.001
Positive social support	6583	22.6	(22.39, 22.82)	23.27	(21.38, 23.91)	0.054
Negative social support	6583	6.80	(6.63, 6.91)	7.56	(6.58, 7.94)	<0.001
Smoking						
Never	5786	84.77%	(83.61, 85.86)	76.91%	(75.61, 81.46)	
Former	113	2.55%	(2.03, 3.19)	8.23%	(6.01, 8.26)	
Current	684	12.68%	(12.7, 13.72)	14.96%	(14.52, 16.47)	

^a^ unweighted counts, ^b^ *p*-value from Chi-square test and *T*-test.

**Table 2:** The characteristics of the variables with functional dentition (n = 6457).

	Functional Dentition						
Variables	No			Yes			^b^ *p*-Value
	^a^ n	Percentage/Mean	95% CI	^a^ n	Percentage/Mean	95% CI	
Age	2361	71.94	(71.42, 72.46)	4222	63.24	(62.89, 63.57)	<0.001
Gender							<0.001
Male	1042	32.7%	(30.8, 34.66)	1864	67.3%	(65.34, 69.2)	
Female	1319	33.74%	(32.1, 45.42)	2358	66.26%	(64.58, 67.9)	0.425
Ethnicity							
White	2311	33.84%	(32.57, 45.14)	4094	66.16%	(64.86, 67.43)	
Non-white	50	19.8%	(14.4, 26.6)	128	80.2%	(73.4, 85.6)	0.0002
Education							
Less than o-level	1241	46.72%	(44.57, 48.88)	1322	53.28%	(51.12, 55.43)	
o-level	630	28.18%	(26.02, 30.45)	1275	71.82%	(69.55, 73.98)	
Higher than A level	490	19.49%	(17.75, 21.36)	1625	80.51%	(78.64, 82.25)	<0.001
Wealth							
Lowest quantile	560	52.85%	(49.23, 56.45)	420	47.15%	(43.55, 50.77)	
Second lowest	538	37.89%	(34.83, 41.04)	638	62.11%	(58.96, 65.17)	
Middle quantile	536	45.84%	(33.12, 38.65)	849	64.16%	(61.35, 66.88)	
Second highest	426	25.04%	(22.82, 27.4)	1068	74.96%	(72.6, 77.18)	
Highest quantile	301	17.69%	(15.77, 19.79)	1247	82.31%	(80.21, 84.23)	<0.001
Positive social support	2361	21.05	(20.69, 21.39)	4222	23.38	(23.11, 23.65)	<0.001
Negative social support	2361	6.44	(6.22, 6.66)	4222	6.94	(6.75, 7.12)	<0.001
Smoking							
Never	1958	31.09%	(29.8, 32.4)	3828	68.91%	(67.6, 70.2)	
Former	30	21.13%	(13.92, 30.75)	83	78.87%	(69.25, 86.08)	
Current	373	50.13%	(45.83, 54.44)	311	49.87%	(45.56, 54.17)	

^a^ unweighted counts, ^b^ *p*-value from Chi-square test and *T*-test.

**Table 3:** Poisson regression models for the association between social support and functional dentition (n = 6457).

Variables	Crude Model		Partially Adjusted Model		Fully Adjusted Model	
	PR	95% CI	PR	95%CI	PR	95%CI
Gender						
Male	1.00	-	1.00	1.00	1.00	-
Female	0.99	(0.96, 1.04)	1.03	(0.99, 1.01)	1.13	(0.99, 1.07)
Ethnicity						
White	1.00	-	1.00	1.00	1.00	-
Non-white	1.19	(1.09, 1.29) ***	1.07	(0.98, 1.01)	1.06	(0.99, 1.13)
Education						
Less than o-level	1.00	-	1.00	1.00	1.00	-
o-level	1.33	(1.26, 1.40) ***	1.11	(1.06, 1.16) ***	1.11	(1.07, 1.17) ***
Higher	1.48	(1.42, 1.56) ***	1.20	(1.15, 1.25) ***	1.19	(1.14, 1.24) ***
Wealth						
Lowest quantile	1.00	-	1.00	1.00	1.00	-
Second lowest	1.30	(1.19, 1.43) ***	1.24	(1.14, 1.34) ***	1.19	(1.10, 1.29) ***
Middle quantile	1.36	(1.24, 1.49) ***	1.39	(1.29, 1.51) ***	1.32	(1.22, 1.42) ***
Second highest	1.57	(1.44, 1.70) ***	1.55	(1.44, 1.67) ***	1.45	(1.35, 1.56) ***
Highest quantile	1.72	(1.59, 1.87) ***	1.63	(1.51, 1.76) ***	1.53	(1.42, 1.65) ***
Age	0.97	(0.96, 0.97) ***	0.97	(0.96, 0.97) ***	0.97	(0.96, 0.97) ***
Positive social support	1.01	(1.01, 1.02) ***	1.01	(1.01, 1.01) ***	1.01	(1.01, 1.01) ***
Negative social support	1.01	(1.01, 1.01) ***	0.99	(0.97, 1.01)	0.99	(0.99, 1.01)
Smoking						
Never	1.00	-			1.00	-
Former	1.14	(1.02, 1.27) **			0.94	(0.84, 1.05)
Current	0.73	(0.67, 0.79) ***			0.74	(0.69, 0.81) ***

Partially adjusted model: adjusted for sociodemographic characteristic (sex, age, ethnicity, wealth, education). Fully adjusted model: further adjusted for smoking. * *p* < 0.05. ** *p* < 0.005. *** *p* < 0.001.

## Data Availability

The original data presented in the study are openly available in the UK Data Service at (https://beta.ukdataservice.ac.uk/datacatalogue/studies/study?id=5050, accessed on 7 February 2025).

## Supplementary Materials

The following supporting information can be downloaded at: https://www.mdpi.com/article/10.3390/dj13120554/s1. Figure S1: A flow chart of the study sample ELSA Wave 7. Table S1: Variance Inflation Factors (VIFs) for Predictors in the Multivariable Model (Unweighted Sample). Table S2: Logistic regression models for the association between social support and functional dentition (n = 6457). Table S3: logistic regression models for the association between social support and functional dentition after excluding edentulous participants (6113).
