# Supplementary material for: Social Support and Functional Dentition Among English Older Adults
Source: Dent J (Basel). 2025 Nov 24;13(12):554. doi: 10.3390/dj13120554 (PMC12731939; doi:10.3390/dj13120554)
Supplement: Supplementary file 1 [file dentistry-13-00554-s001.zip › dentistry-3906799-supplementary.pdf]

Supplementary Figure S1 – A flow chart of the study sample ELSA Wave 7.

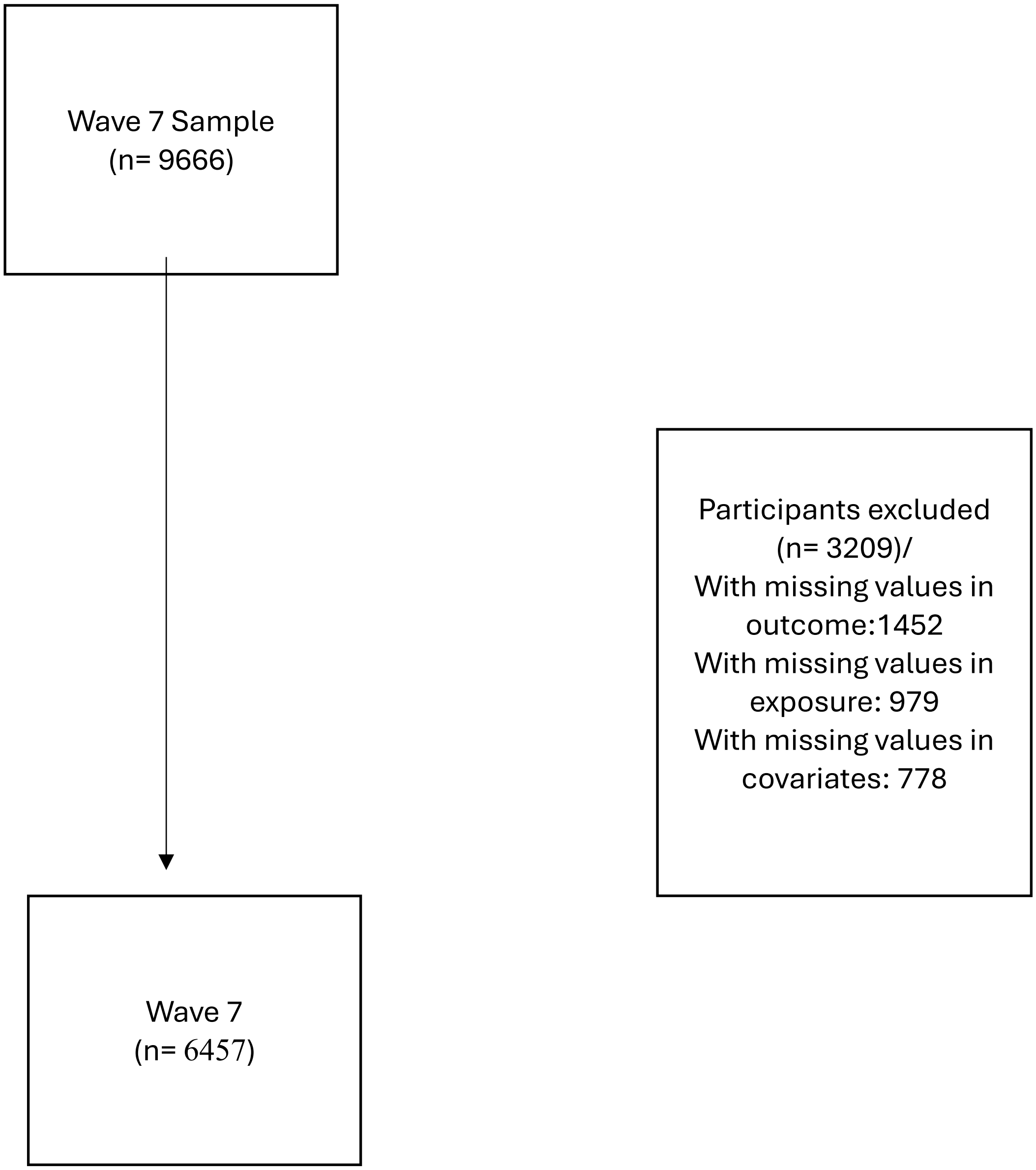

Supplementary Table S1 - Variance Inflation Factors (VIFs) for Predictors in the Multivariable Model (Unweighted Sample).

**Variables**	**VIF**	**1/VIF**
Age	16.28	0.061420
Positive social support	9.91	0.100862
Sex	9.89	0.101090
Wealth	7.49	0.133562
Negative social support	2.78	0.360335
Education	2.44	0.410243
Smoking	1.16	0.865246

Supplementary Table S2 –Logistic regression models for the association between social support and functional dentition (n= 6,457).

Variables		Crude Model		Partially adjusted Model		Fully adjusted Model	
		OR	95% CI	OR	95%CI	OR	95%CI
Gender							
	Male	1.00	-	1.00	-	1.00	-
	Female	0.95	(0.85, 1.07)	1.16	(1.01, 1.31)*	1.14	(1.00, 1.30)*
Ethnicity							
	White	1.00	-	1.00	-	1.00	-
	Non-white	2.07	(1.40, 3.05)***	1.72	(1.15, 2.56)**	1.63	(1.08, 2.46)**
Education							
	Less than o-level	1.00	-	1.00	-	1.00	-
	o-level	2.23	(1.94, 2.57)***	1.33	(1.14, 1.55)***	1.35	(1.15, 1.57)***
	Higher	3.62	(3.14, 4.18)***	2.12	(1.80, 2.49)***	2.08	(1.76, 2.45)***
Wealth							
	Lowest quantile	1.00	-	1.00	-	1.00	-
	Second lowest	1.84	(1.51, 2.24)***	1.80	(1.45, 2.25)***	1.58	(1.27, 1.97)***
	Middle quantile	2.01	(1.66, 2.42)***	2.57	(2.07, 3.18)***	2.20	(1.78, 2.72)***
	Second highest	3.36	(2.77, 4.06)***	3.95	(3.19, 4.89)***	3.26	(2.63, 4.04)***
	Highest quantile	5.21	(4.27, 6.37)***	5.19	(4.10, 6.56)***	4.28	(3.38, 5.41)***
Age		0.91	(0.91, 0.92)***	0.91	(0.90, 0.91)***	0.90	(0.89, 0.91)***
Positive social support		1.04	(1.03, 1.05)***	1.02	(1.01, 1.03)***	1.02	(1.01, 1.03)***
Negative social support		1.02	(1.01, 1.03)**	0.98	(0.97, 0.99)**	0.98	(0.97, 0.99)**
Smoking							
	Never	1.00	-	1.00	-	1.00	-
	Former	1.68	(1.01, 2.80)*			0.92	(0.48, 1.74)
	Current	0.45	(0.37, 0.54)***			0.35	(0.29, 0.43)***

Partially adjusted model: adjusted for sociodemographic characteristic (sex, age, ethnicity, wealth, education).
Fully adjusted model: further adjusted for smoking.
*P<0.05.
**p<0.005.
***p< 0.001.

Supplementary Table S3 – logistic regression models for the association between social support and functional dentition after excluding edentulous participants (6,113).

Variables	Fully adjusted Model	
	OR	95%CI
Gender		
Male	1.00	-
Female	1.18	(1.04, 1.33)**
Ethnicity		
White	1.00	-
Non-white	1.63	(1.08, 2.46)**
Education		
Less than o-level	1.00	-
o-level	1.20	(1.04, 1.39)*
Higher	1.86	(1.59, 2.17)***
Wealth		
Lowest quantile	1.00	-
Second lowest	1.32	(1.07, 1.62)**
Middle quantile	1.78	(1.45, 2.17)***
Second highest	2.44	(1.98, 3.00)***
Highest quantile	3.18	(2.56, 3.96)***
Age	0.92	(0.89, 0.91)***
Positive social support	1.01	(1.01, 1.02)**
Negative social support	0.98	(0.96, 0.99)**
Smoking		
Never	1.00	-
Former	1.02	(0.62, 1.69)
Current	0.38	(0.31, 0.46)***

*P<0.05.
**p<0.005.
***p< 0.001.
